# Absolute Oral Bioavailability of Creatine Monohydrate in Rats: Debunking a Myth

**DOI:** 10.3390/pharmaceutics10010031

**Published:** 2018-03-08

**Authors:** Eman A. Alraddadi, Ryan Lillico, Jonathan L. Vennerstrom, Ted M. Lakowski, Donald W. Miller

**Affiliations:** 1 Department of Pharmacology and Therapeutics, The Rady Faculty of Health Sciences, University of Manitoba, Winnipeg, MB R3E 3J7, Canada; alraddae@myumanitoba.ca; 2 Pharmaceutical Analysis Laboratory, College of Pharmacy, The Rady Faculty of Health Sciences, University of Manitoba, Winnipeg, MB R3E 0T5, Canada; umlillic@myumanitoba.ca (R.L.); ted.lakowski@umanitoba.ca (T.M.L.); 3 Department of Pharmaceutical Sciences, College of Pharmacy, University of Nebraska Medical Center, Omaha, NE 68198-6125, USA; jvenners@unmc.edu

**Keywords:** creatine monohydrate, pharmacokinetics, oral bioavailability, LC-MS/MS, physiology-based pharmacokinetic modeling

## Abstract

Creatine is an ergogenic compound used by athletes to enhance performance. Supplementation with creatine monohydrate (CM) has been suggested for musculoskeletal and neurological disorders. Until now, little is known about its pharmacokinetic profile. Our objective was to determine the oral bioavailability of CM and the influence of dose on oral absorption. Rats were dosed orally with low dose (10 mg/kg) or high dose (70 mg/kg) ^13^C-labeled CM. Blood samples were removed at various time points. Muscle and brain tissue were collected at the conclusion of the study. Plasma and tissue levels of ^13^C-labeled creatine were determined using liquid chromatography-tandem mass spectrometry (LC-MS/MS). Physiologically based pharmacokinetic (PBPK) models of CM were built using GastroPlus™. These models were used to predict the plasma concentration–time profiles of creatine hydrochloride (CHCL), which has improved aqueous solubility compared to CM. Absolute oral bioavailability for low dose CM was 53% while high dose CM was only 16%. The simulated C_max_ of 70 mg/kg CHCL was around 35 μg/mL compared to 14 μg/mL for CM with a predicted oral bioavailability of 66% with CHCL compared to 17% with CM. Our results suggest that the oral bioavailability of CM is less than complete and subject to dose and that further examination of improved dosage formulations of creatine is warranted.

## 1. Introduction

Creatine is a naturally occurring guanidine compound mostly found in skeletal muscles (95%) [1], with smaller amounts found in brain, kidneys, liver, and testes [2]. In addition to the synthesis in the liver and kidneys, creatine can be obtained exogenously through the diet especially in protein-based foods such as meat, fish, and nuts [2]. Creatine is part of the adenosine triphosphate (ATP)/phosphocreatine (PCr) phosphogen energy system, and plays a critical role in providing a readily available source of phosphate for the replenishment of ATP [3]. Creatine dietary supplements, mostly in the form of creatine monohydrate (CM), have been widely used as an ergogenic aid by athletes and bodybuilders to enhance exercise and performance [4]. In addition, creatine supplementation has been shown to be beneficial in the treatment of certain diseases, such as those involving muscular atrophy or fatigue secondary to impaired energy production [2]. Also, recent evidence indicates creatine may be useful in the treatment of many neurodegenerative disorders including Huntington’s disease, Alzheimer’s disease, and Parkinson’s disease [5,6,7,8,9,10].

Dietary supplementation with CM for improved athletic performance typically involves a loading dose of 20 g per day over a five day period, followed by a daily maintenance dose of three to five grams [2]. This regimen has been reported to result in an approximately 20% increase in intramuscular creatine levels [11] and to enhance strength and improve recovery time in a variety of performance conditions [11,12,13]. Dietary supplementation with CM for therapeutic indications typically require daily doses that exceed 20 g [6]. Indeed, in clinical studies of Huntington’s disease, patients received as much as 30 g daily doses of CM [6]. Given the aqueous solubility of CM, which is around 18 mg/mL [14], doses above three to five grams are likely administered as a suspension. Thus dietary supplementation with CM can vary greatly in dosage and dosage form depending on the indication and desired effect.

While it is widely assumed that CM is 100% bioavailable and has complete or nearly complete absorption in the gastrointestinal tract [15], little scientific evidence for this is available. Previous in vitro studies of CM permeability in the Caco-2 model [16,17] suggest that the passage of creatine across the intestinal epithelium is limited. These studies, together with the observation that large doses are required to achieve the desired performance and therapeutic effects of creatine, suggest that the oral bioavailability of CM is incomplete. The objective of the current study was to examine the oral bioavailability, pharmacokinetic (PK) properties and tissue distribution of CM following single oral dose administration in rats. As single doses in humans can vary from one gram to more than 20 g [2], the potential influence of dose on oral absorption was also examined. Our results indicate that the oral bioavailability of CM is dose-dependent with the low dose having greater bioavailability than high dose. Given the anticipated concentrations of CM in the intestinal lumen, the reduced bioavailability with high dose CM is likely due to solubility issues rather than potential transport or carrier interactions at the site of absorption. This was further confirmed using physiological-based PK (PBPK) modeling of CM and other more soluble creatine supplements.

## 2. Materials and Methods 

### 2.1. Materials

Sprague–Dawley rats were purchased from Charles River Laboratories (Wilmington, MA, USA). Creatine-(guanidino-^13^C) monohydrate (CM-^13^C) (product number 569925) was obtained from Sigma Aldrich (St. Louis, MO, USA). Creatine (methyl-d_3_) (product number DLM-1302-0.25) was obtained from Cambridge Isotope Laboratories, Inc. (Tewksbry, MA, USA). Catheter locking solution was purchased from SAI infusion technologies (Lake Villa, IL, USA). All other chemicals were purchased from Sigma-Aldrich unless otherwise stated.

### 2.2. PK Study in Rats

The study was approved by the University of Manitoba Animal Care Committee (Protocol 14-010; 27 January 2014) and was performed in accordance with the Canadian Council on Animal Care guidelines. Twelve adult male Sprague–Dawley rats weighing 280–310 g were anesthetized with isoflurane and implanted with jugular vein catheters. The catheters were externalized and secured on the dorsal side of the neck. Rats were allowed to recover and acclimatize for at least one week before the start of the study. During this period, the catheters were flushed with heparinized saline every other day. In addition, the rats had free access to food and water and were kept in a facility with a 12 h light/dark cycle, controlled humidity (55 ± 5%), and controlled temperature (21 ± 2 °C). Food was withheld 12 h prior to the PK study.

Rats were given either low dose (10 mg/kg) or high dose (70 mg/kg) of CM-^13^C, dissolved in normal saline solution. Doses (1 mL/kg) were administered via oral gavage. A separate treatment group received 10 mg/kg CM-^13^C by bolus intravenous (iv) injection. Serial blood samples (0.2 mL) were collected at various time points (0–240 min) following oral and iv administration of CM-^13^C in tubes containing citrate buffer. Following the last blood sampling (i.e., 240 min after creatine administration), rats were anesthetized and euthanized by decapitation, muscle and brain tissue samples were collected and immediately freeze-clamped in liquid nitrogen. Following liquid nitrogen freezing, tissue was weighed, and stored at −80 °C. Plasma fractions were immediately obtained from blood samples by centrifugation (15 min at 2000× *g*) and stored at −80 °C until further analysis by liquid chromatography-tandem mass spectrometry (LC-MS/MS).

Plasma data were subjected to non-compartmental PK analysis using Excel. The initial serum concentration (C_p0_) of creatine-^13^C was obtained directly from the log concentration–time curve. The maximum plasma concentration (C_max_) and time taken to reach C_max_ (T_max_) were estimated directly from the plasma concentration–time curves. The elimination rate constant (λ_z_) was determined by linear regression analysis of the terminal phase of the plasma concentration (log scale)–time curve. The total area under the plasma concentration–time curve (AUC_0–t_) was calculated using the linear trapezoidal rule. The AUC from 0 to infinity (AUC_0–∞_) was calculated using the formula: AUC_0–t_ + C_last_/λ_z_; where C_last_ is the last measurable nonzero plasma concentration. Absolute oral bioavailability (F) was calculated using the formula: (AUC_oral_/AUC_IV_) × (Dose_IV_/Dose_oral_). The half-life (T_1/2_) was calculated using the formula: T_1/2_ = In2/λ_z_. Clearance (CL) was calculated as CL = dose/AUC_0–∞_. The apparent volume of distribution (V_d_) was calculated as V_d_ = CL/λ_z_.

### 2.3. Sample Preparation

#### 2.3.1. Plasma and RBC Samples Preparation

Blood samples were centrifuged for 10 min at 2000× *g* and the plasma and cell fractions collected and stored at −80 °C. For LC-MS/MS analysis, 10 μg/mL of the internal standard, creatine-d_3,_ dissolved in citrate buffer (0.13 g citrate/mL distilled water, pH 4.3), was added to 100 μL of plasma from each sample. Then, 1 mL of cold acetonitrile (with 0.3% Formic acid, pH 3) was added to each sample to precipitate protein. The samples were vortexed for 2 min and centrifuged at 15,000× *g* for 5 min. The supernatant was transferred to new tubes and evaporated to dryness using a Savant SPD1010 SpeedVac Concentrator (Thermo Fisher Scientific, Inc., Asheville, NC, USA) at 45°. The cell fraction from the collected blood was thawed and the lysed cells were then processed as described above for plasma samples.

#### 2.3.2. Brain and Muscle Samples Preparation

Brain and muscle tissue were homogenized in citrate buffer at a concentration of 1.3 g tissue/7 mL citrate buffer using electric homogenizer. Then, 10 μg/mL of the internal standard was added to 100 μL of the samples. After vortex, 1 mL of ice-cold acetonitrile was added and the samples were centrifuged at 15,000 rpm for 5 min at 4 °C. Supernatants were collected and transferred to a clean tube. Samples were then dried using SpeedVac.

### 2.4. LC-MS/MS Analysis

The analytical system used was a Shimadzu LCMS-8040 triple-quadrupole mass spectrometer; LC-MS/MS (Shimadzu, Kyoto, Japan) coupled to a Nexera ultra high performance liquid chromatograph (Shimadzu, Kyoto, Japan) and data was analyzed using Shimadzu LabSolutions software (Version 5.72). The LC-MS/MS was operated in DUIS mode (ESI/APCI) using multiple reaction monitoring (MRM). The LC-MS/MS conditions consisted of a desolvation line temperature of 250 °C and heating block temperature of 400 °C. Nebulizing gas flow was 2 L/min and drying gas was 15 L/min.

Tissue and plasma samples were analyzed for creatine-^13^C. The analytical column used was a Primesep 200 (3 μm, 2.1 × 100 mm) (SIELC Technologies, Wheeling, IL, USA) mixed function cation exchange column. The mobile phase consisted of a pH gradient with mobile phases A (0.05% aqueous formic acid) and B (1% formic acid in acetonitrile). A linear gradient was applied from 0% to 85% B over 4 min, held at 85% B for 2 min followed by a step down to 0% B and held for 4 min to recondition and equilibrate the column prior to the next injection. The total flow rate of the system was 0.4 mL/min and the column oven was set at 40 °C. The following transitions were monitored in positive MRM mode: *m*/*z* 133.1 > 90.1 (collision energy (CE) of 15 eV) for creatine-^13^C and 135.1 > 93.1 (CE of 15 eV) for creatine-d_3_.

#### 2.4.1. Stock and Working Standard Solutions

All stock solutions were prepared at 1000, 100, 10 and 1 μg/mL concentrations in citrate buffer (0.13 g/mL) at pH 4.3. These solutions were stored at −20 °C and remade after 3 freeze–thaw cycles. Calibration standards containing 10 μg/mL internal standard in rat plasma, muscle or brain homogenate were prepared from stock solutions by dilution to a series of concentrations as 0.01, 0.05, 0.1, 0.5, 1.0, 5.0, 10 and 50 μg/mL. Plasma and tissue samples from untreated rats were prepared containing 10 μg/mL internal standard to evaluate background signal.

#### 2.4.2. Sample Preparation for Standards

The working standards were added to 100 μL plasma, muscle homogenate or brain homogenate (homogenized at 1.3 g tissue/7 mL citrate buffer) containing 10 μg/mL internal standard in microcentrifuge tubes for concentrations described above. Cold acetonitrile with 0.3% formic acid (1 mL at −20 °C) was promptly added to the samples to precipitate proteins. The samples were vortexed for 2 min and centrifuged at 15,000× *g* for 5 min. The supernatant was transferred to new tubes and evaporated to dryness using SpeedVac at 45 °C. The dried samples were reconstituted in 50 μL of 0.05% aqueous formic acid and 3 μL were injected into the LC-MS/MS system.

### 2.5. Physiologically Based Pharmacokinetic Modeling (PBPK)

All PBPK models and simulations were performed using GastroPlus, version 9.0 (Simulations Plus Inc., Lancaster, CA, USA). This module simulates and predicts PK profiles of compounds using input parameters based on the physicochemical properties (e.g., solubility, LogP, pKa) of the compound and species-specific physiological disposition properties (e.g., V_d_, blood flow and renal and metabolic clearance rates). The modeling program consisted of various tissue compartments, including the heart, lung, liver, spleen, gastrointestinal tract, adipose tissue, skeletal muscle, brain, kidney, skin, reproductive organs linked together by venous and arterial blood circulation. Physiological parameters including tissue volume and blood flow and vascular permeability were set within the software for the species of interest. The PBPK model was used to simulate plasma concentration–time curves and predict tissue distribution and PK parameters, such as C_max_, T_max_, V_d_ CL and AUC_,_ for both CM and creatine hydrochloride (CHCl).

### 2.6. Statistics

All data were expressed as mean ± standard error of the mean (SEM). Statistical significance was evaluated using one-way ANOVA with Tukey post-hoc comparison of the means.

## 3. Results

### 3.1. LC-MS/MS Assay

Assay limits and accuracy were determined by inter-day analysis of standard curves prepared in rat plasma (*n* = 4). The lower limit of quantitation (LLOQ) was determined to be 0.5 μg/mL as the lowest point on the curve to achieve a coefficient of variation (CV) no higher than 20%. This was calculated to be 0.551 ± 0.098 μg/mL (CV of 17.8%). The curve was linear between 0.5 and 50 μg/mL with no point other than LLOQ exceeding 15% CV. Quality control samples of 2.5 and 10 μg/mL were measured during analysis and their recoveries were 98.0 ± 3.76% and 99.6 ± 1.09% respectively. Unknown samples exceeding 50 μg/mL were diluted using mobile phase containing 10 μg/mL internal standard and calculated based on the dilution factor. All back-calculated samples were within 15% agreement with their original extrapolated values, suggesting linearity beyond 50 μg/mL.

**Figure 1:**
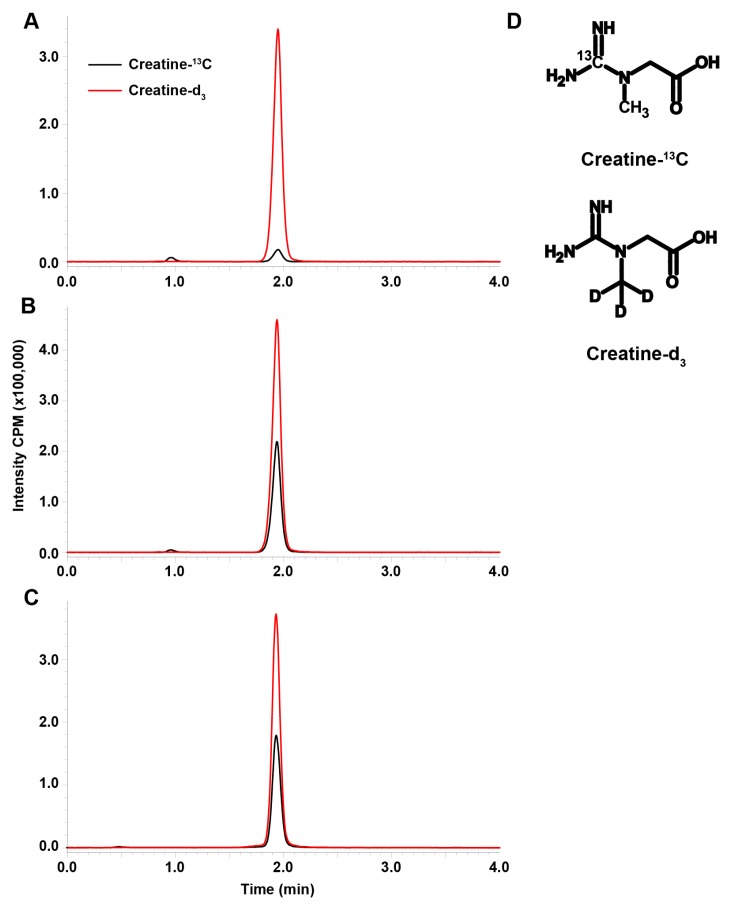
The representative chromatograms of creatine-^13^C (black) and creatine-d_3_ (red) in blank plasma (**A**), a 10 μg/mL standard sample of creatine-^13^C in plasma (**B**), and an unknown rat plasma sample in similar range as the standard which we measured as 10.7 μg/mL creatine-^13^C (**C**). The assay was developed for the simultaneous measurement of creatine-guanidino-^13^C and creatine-methyl-d_3_ (**D**). Intensity is in counts per minute (CPM).

Blank plasma containing 10 μg/mL internal standard (Figure 1A) showed trace amounts of creatine-^13^C from endogenous creatine due to the natural abundance of ^13^C. This was accounted for since the curve was generated in plasma; however, this is a limitation to the assay with respect to the LLOQ in comparison to other LC-MS/MS assay for creatine [18,19]. Standard in plasma at 10.0 μg/mL and an unknown measured to be 10.7 μg/mL (Figure 1B,C respectively) show consistency in retention time and ionization demonstrating the robustness of the assay. The choice of stable isotope labeled internal standards was made based on the similarity of the internal standard to the analyte (Figure 1D).

### 3.2. Plasma Kinetics and Oral Bioavailability of Low Dose and High Dose CM

**Figure 2:**
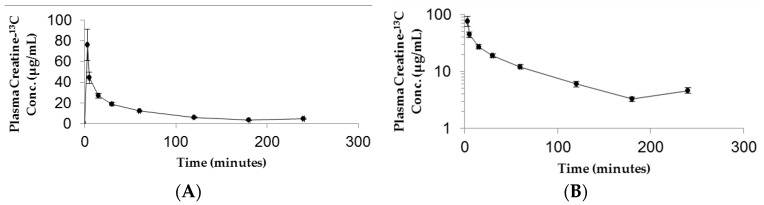
Plasma ^13^C-creatine concentration vs time curves following intravenous administration of CM-^13^C in adult male rats. The curves are shown in both linear (**A**) and semi-log (**B**) formats. Values represent the mean ± SEM. *n* = 4 rats.

**Figure 3:**
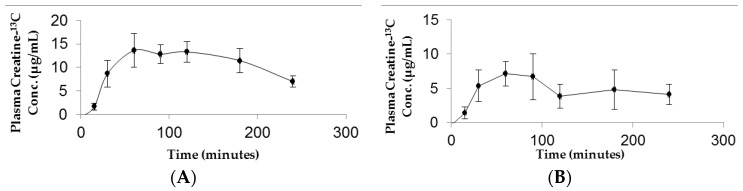
Plasma concentration–time curve following administration of (**A**) high dose (70 mg/kg) or (**B**) low dose (10 mg/kg) oral CM-^13^C. Values represent the mean ± SEM. *n* = 4 rats.

The resulting plasma concentration–time curve for CM-^13^C following iv bolus injection is shown in Figure 2. The peak plasma concentration of creatine-^13^C was 76.18 ± 15.22 μg/mL (Figure 2A). Removal of creatine-^13^C from the plasma compartment following iv bolus administration indicated a multiple compartment PK model with rapid distribution phase followed by a slow terminal elimination phase (Figure 2B). The concentration–time curves for oral high dose (70 mg/kg) and low dose (10 mg/kg) CM-^13^C are shown in Figure 3A,B, respectively. While the T_max_ were similar for both high and low dose CM (60 min) following oral administration, C_max_ were 13.59 ± 3.57 and 7.14 ± 1.79 μg/mL, respectively. The AUC_0–∞_ for oral high dose (70 mg/kg), low dose (10 mg/kg), and iv CM-^13^C were 2501.33 ± 378, 1139.5 ± 488, and 2450.01 ± 110 μg·h/mL, respectively. The absolute oral bioavailability for low dose CM was 53.22 ± 11.2% while high dose CM was only 15.69 ± 3.4%. While ^13^C-labeled-creatinine (a metabolite of creatine) was also analyzed in these studies, the amount of ^13^C-creatinine was unchanged from baseline values at all time points examined (data not shown).

### 3.3. Tissue Distribution Following Low Dose and High Dose CM

**Figure 4:**
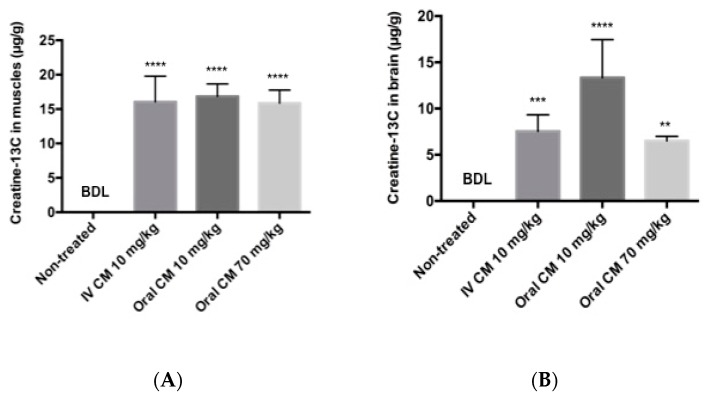
(**A**) Muscle and (**B**) brain concentrations of Creatine-^13^C 4 h after administration of CM-^13^C. Values represent the mean ± SEM. Creatine-^13^C content in muscle and brain samples from non-treated rats was below detection limits (0.5 μg/g tissue). *n* = 4 rats. ** *p* < 0.01, *** *p* < 0.001, **** *p* < 0.0001 compared to levels in non-treated group. BDL = below detection limit.

Tissue distribution of creatine-^13^C following oral and iv injection of CM-^13^C was quantified using LC-MS/MS. Tissue accrual was the highest in the muscle samples reaching approximately 16 μg/g at four hours following either oral or iv CM-^13^C (Figure 4A). While increases in creatine-^13^C were also detected in the brain, the levels were about a third of that observed in muscle (Figure 4B). The creatine-^13^C content in the RBC samples following oral administration showed little change of baseline levels taken prior to administration of CM-^13^C. However, RBC levels of creatine-^13^C following iv injection peaked at three minutes and was at or near baseline levels at four hours. Muscle and brain concentrations of creatine-^13^C were normalized to blank muscle and brain values.

### 3.4. Modeling of Creatine Pharmacokinetics in Rats

**Figure 5:**
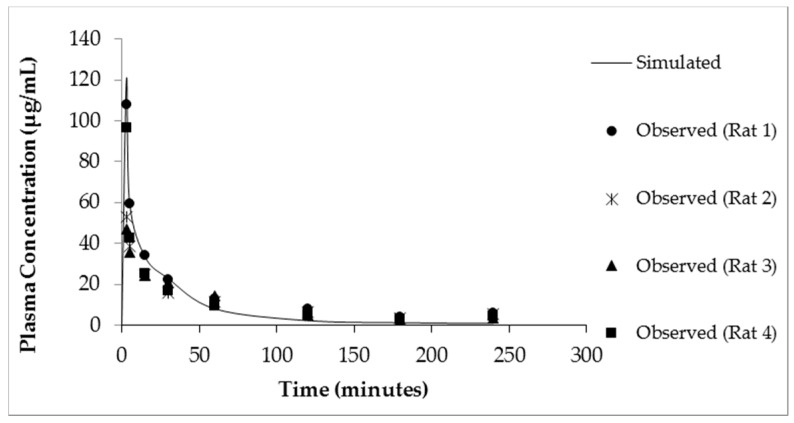
Simulated (solid line) and observed plasma creatine concentration–time curves after a single dose of iv CM (10 mg/kg).

**Figure 6:**
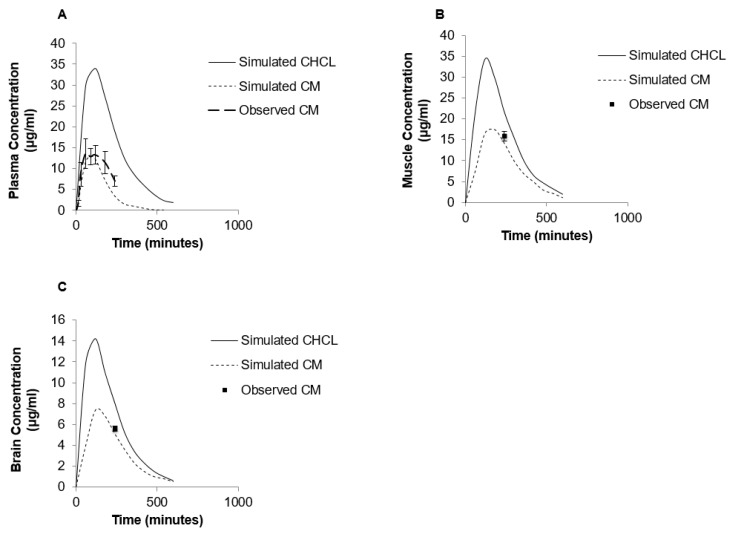
Simulated levels of creatine in plasma (**A**), muscle (**B**) and brain (**C**) following single 70 mg/kg oral dose of CHCl (solid line) or CM (dashed line) using GastroPlus™ modeling software compared to observed values. Note that due to differences in aqueous solubility the CM dosage was a suspension, while CHCl was completely in solution.

The simulated plasma concentration–time curves following administration of either a bolus iv injection of CM (10 mg/kg) or oral suspension of CM (70 mg/kg) in rats are shown in Figure 5 and Figure 6A, respectively. The simulation curves generated using GastroPlus™ were compared to observed values. The predicted PK parameters from the modeling program and the observed values are listed in Table 1. For the bolus iv injection route there was very good agreement between observed and PBPK model-simulated values with R-squared value (*R*^2^) = 0.99. The same PBPK model displayed some divergence from observed values in high dose oral absorption (especially at the later time points) (Figure 6A). However, the *R*^2^ value for oral administration route was still 0.84 (Table 1). Considering the variability within the data set, the model provided reasonable approximations of PK parameters for oral dosing of CM (Table 1).

The PBPK model was also used to predict the impact of other creatine salt forms on plasma and tissue levels of creatine. For these simulations, CM was compared to CHCl, a newer salt form of creatine with higher aqueous solubility (approximately 700 mg/mL) [14]. The predicted PK profile following oral administration of 70 mg/kg dose of CHCl compared to a similar dose of CM is shown in Figure 6. The predicted C_max_ of CHCl was around 35 μg/mL compared to 14.07 μg/mL for CM, and the predicted oral bioavailability was 66% compared to approximately 16.8% with CM. The differences in plasma creatine levels with CM and CHCl were attributed to the enhanced aqueous solubility of CHCl. In addition to increased plasma levels of creatine, an increase in tissue levels of creatine were also predicted with the PBPK model of CHCl compared to CM (Figure 6B,C). The simulated muscle concentrations of creatine peaked at approximately 34 μg/mL for CHCl compared to approximately 17 μg/mL with same dose of CM (Figure 6B). For the brain, estimated levels of creatine reached approximately 14.5 μg/mL with CHCl compared to approximately 8 μg/mL with CM (Figure 6C).

## 4. Discussion

In this paper, we examined the PK profile and oral bioavailability of CM. While there has been much research devoted to understanding the uptake of CM into muscle cells and to explore the effects of CM on exercise and performance, few studies have examined the oral bioavailability of CM supplements. There is an assumption that creatine supplements, of which CM is the most widely used, are completely absorbed from the gastrointestinal tract. This assumption is based on the very early studies of Chanutin and colleagues [20], and more recent studies of Deldicque et al. [15], in which low doses of CM (two grams) were given orally to healthy individuals and the resulting levels of creatine in tissue and the increase of excreted creatinine was used to assess the bioavailability of CM. Both studies claimed a complete absorption of orally administered creatine on the basis of increased creatine in blood and tissue and the absence of creatine or creatinine observed in fecal samples. However, this approach assumes any creatinine observed in the urine reflects systemically absorbed creatine and neglects to account for potential creatine utilization by the bacterial flora in the gastrointestinal tract [21]. In addition, potential issues such as gastric degradation, site dependent intestinal absorption and incomplete dissolution of creatine solid dosage forms, outlined by McCall and Persky [22], are all likely to result in less than complete absorption of creatine from the gastrointestinal tract.

To accurately determine oral bioavailability, an iv administration treatment arm is required. In the present study, iv administration of CM-^13^C resulted in a rapid distribution of creatine out of the central (blood) compartment and a slower terminal elimination phase. This is consistent with known distribution of creatine to skeletal muscle, brain and other tissues [23]. There was a slight increase in plasma creatine observed at the last time point sampled following iv administration of CM-^13^C. Multiple peaking PK can occur due to a variety of both formulation and physiological factors [24]. For the iv administration route, the most likely contributor to multiple peaking phenomena would be enterohepatic recycling [24]. As the physicochemical properties and metabolic pathway for creatine are not supportive of biliary secretion [2], it is unlikely that enterohepatic recycling of creatine is occurring in the present study. Given the rapid distribution of creatine into skeletal muscle and relatively large depot site that it represents, the plasma creatine levels observed likely reflect the slow and sustained release of creatine from these deep tissue sites.

Oral administration of CM-^13^C resulted in dose-dependent increases in plasma creatine. Maximal plasma ^13^C-creatine levels increased 10 to 25-fold from baseline levels (<0.5 μg/mL). The plasma creatine profile observed in the present study was similar to those previously reported [15,25,26,27]. Despite the substantial increase in plasma creatine, there was no detectable increase in ^13^C-creatinine in the present study. While increases in plasma creatinine following oral creatine administration have been observed [27], others have reported no change in blood creatinine [26]. It should be noted that those studies reporting increases in plasma creatinine following oral creatine administration were using either large doses (20 g) of creatine [27] or extended multi-day exposure [15,25]. Even under these conditions the increases in plasma creatinine were small in comparison to the increases in plasma creatine; thus the conversion of creatine to creatinine was not considered to play a significant role in creatine elimination [22,27].

With both the iv or oral PK profiles, it was possible to determine the absolute oral bioavailability of CM by comparing the resulting AUC_0–∞_ for CM-^13^C when given by the oral route. For the low oral dose, where there was complete dissolution of CM, the oral bioavailability was 53%. While such absorption is substantial, it is far from complete. In comparison, for the high (70 mg/kg) dose, were the administered CM-^13^C was as a suspension, the oral bioavailability was 16%. The conditions selected for the high dose CM PK studies reported here were designed to more closely represent the dosing used in therapeutic applications where large daily doses (30 g or more) of CM were administered in suspension form. These data for both low and high dose oral CM administration suggest that CM oral bioavailability is not complete. While these findings represent a significant departure from the relatively complete oral absorption of CM claimed in earlier reports [15,20,26], it should be noted that in none of these previous studies was an iv treatment arm included to accurately assess bioavailability.

While suggested in various human studies [28,29], this study is the first to assess dose-dependent changes in oral bioavailability of CM. The dose-dependent CM oral bioavailability could be due to saturation of transport driven absorption processes in the intestine or incomplete dissolution of solid dosage forms typically used in CM supplementation. Given the transporter kinetics [30], it is likely that concentrations of creatine in the gastrointestinal tract will be well above those required for saturation of the creatine transporter at even the lowest doses of CM administered. The more plausible reason for potential dose dependency with CM supplementation is the incomplete dissolution of CM under most dosing conditions. Given the low aqueous solubility of CM, which is around 18 mg/mL [14], and the high doses required for CM supplementation (i.e., around 20+ g/day), a suspension is the likely form of CM being administered. In the present study, the 70 mg/kg dose was administered as a suspension. Under these conditions, oral bioavailability was even less than observed with the 10 mg/kg dose. Another indication that the reduction in oral bioavailability of the high dose CM was likely due to incomplete solubilization, was the PBPK modeling performed with CHCl, a creatine salt with improved aqueous solubility [14]. While the model simulations predicted 17% oral bioavailability with the 70 mg/kg dose of CM, the same dose of CHCl resulted in a predicted oral bioavailability around 66%, which is comparable with the bioavailability observed following oral administration of low dose CM-^13^C.

Of the tissue compartments examined, skeletal muscle accumulated the most creatine-^13^C, followed by the brain and the blood cells. The creatine distribution in the various tissues following oral and iv exposure was consistent with previous studies showing highest accumulation in skeletal muscle (up to 90%) and brain [23]. As creatine in the bloodstream is taken up by cells through a specific plasma membrane creatine transporter (CT1) [31], the tissue concentration of creatine may also be dependent on CT1 transport. CT1 is a saturable sodium-dependent membrane transporter that is expressed predominantly in the skeletal muscles, kidney, heart, brain, colon, and testes [32,33,34]. In the brain, the CT1 gene is expressed in the neurons, oligodendrocytes, and in the brain microvessel endothelial cells at the blood–brain barrier (BBB) [35,36].

Tissue accumulation of creatine through the CT1 is a saturable process [37]. Michaelis constant (K_m_) values of CT1 and serum creatine concentrations in rats are around 22–46 μM and 140 μM, respectively [2,37]. Based on blood concentrations of creatine, it is apparent from these K_m_ values that CT1 is working close to saturation suggesting a possible limitation of creatine accumulation [37]. For example, in unsupplemented rats, normal plasma levels of creatine are around 140–150 μM and with K_m_ values of 22–46 μM, suggesting that the transporter is working near saturation and any further increase in the plasma concentrations of creatine will most likely saturate the transporter limiting the uptake of creatine.

There is increasing interest in different salt forms of creatine with improved solubility parameters over CM [14,38]. One of the newer salt forms of creatine, CHCl, has an aqueous solubility of around 700 mg/mL [14]. The markedly enhanced solubility of CHCl to that of CM suggests that improved oral absorption and more efficient dosing formulations should be possible. Indeed, based on the PBPK model simulations, substantial increases in blood and tissue levels of creatine are likely following oral supplementation with CHCl compared to CM. While our model predictions will require additional validation, there was reasonable agreement of the PBPK model to the observed iv and oral CM (*R*^2^ of 0.99 and 0.84, respectively). PBPK modeling is increasingly being used for retrospective and prospective simulation and prediction of animal and human PK [39,40,41,42,43,44,45,46]. It should also be noted that our predictions of improved oral absorption and PK properties with more soluble creatine salt forms are consistent with previous studies comparing creatine citrate and creatine pyruvate salts. In these studies, Jager et al. compared the oral bioavailability of creatine citrate and creatine pyruvate to that of CM. They found that creatine pyruvate, which has around eight-fold higher aqueous solubility than CM, had a significant (approximately 25%) increase in oral bioavailability compared to the latter [38]. In addition to providing further evidence of less than complete oral bioavailability for CM, such findings suggest that creatine salts with improved aqueous solubility and oral absorption characteristics could provide improvements over CM in therapeutic applications requiring high doses of creatine [47].

## 5. Conclusions

Our results suggest that the oral bioavailability of CM is less than complete and is dose-dependent. As most therapeutic indications require rather large doses (>150 mg/kg), the actual bioavailability of such doses may be limited by either physiological and/or formulation factors. Examination of the PK of CM at therapeutically relevant doses is warranted. These studies also suggest that newer forms and dosage formulations of creatine might be superior to CM, but further studies comparing newer formulations to CM will be required.

## Figures and Tables

**Table 1:** Comparison between predicted CM pharmacokinetic parameters and observed values following iv and oral dose of CM.

Parameter	IV Injection of CM (10 mg/kg)		Oral Suspension of CM (70 mg/kg)	
	Simulated	Observed	Simulated	Observed
C_max_ (μg/mL)	122	76.18 ± 15.23	14.07	13.72 ± 3.57
T_max_ (min)	3	3	87	60
V_d_ (L/kg)	0.208	0.304 ± 0.087	-	-
T_1/2_ (min)	64.08	69.3 ± 3.7	-	-
CL (L/hr)	0.039	0.051 ± 0.01	-	-
AUC_0–∞_ (μg·h/mL)	2279.36	2450.01 ± 110	2286.1	2501.33 ± 378
F (%)	-	-	16.8	15.69 ± 4.3
*R*^2^ value (model vs. observed)	0.99	-	0.84	-

## References

[1] Hall M., Trojian T.H. (2013). Creatine supplementation. Curr. Sports Med. Rep..

[2] Persky A.M., Brazeau G.A. (2001). Clinical pharmacology of the dietary supplement creatine monohydrate. Pharmacol. Rev..

[3] Wallimann T., Wyss M., Brdiczka D., Nicolay K., Eppenberger H.M. (1992). Intracellular compartmentation, structure and function of creatine kinase isoenzymes in tissues with high and fluctuating energy demands: The ‘phosphocreatine circuit’ for cellular energy homeostasis. Biochem. J..

[4] Williams M.H., Branch J.D. (1998). Creatine supplementation and exercise performance: An update. J. Am. Coll. Nutr..

[5] Beal M.F. (2011). Neuroprotective effects of creatine. Amino Acids.

[6] Rosas H.D., Doros G., Gevorkian S., Malarick K., Reuter M., Coutu J.P., Triggs T.D., Wilkens P.J., Matson W., Salat D.H. (2014). PRECREST: A phase II prevention and biomarker trial of creatine in at-risk Huntington disease. Neurology.

[7] Brewer G.J., Wallimann T.W. (2000). Protective effect of the energy precursor creatine against toxicity of glutamate and beta-amyloid in rat hippocampal neurons. J. Neurochem..

[8] Vis J.C., de Boer-van Huizen R.T., Verbeek M.M., de Waal R.M.W., ten Donkelaar H.J., Kremer B. (2004). Creatine protects against 3-nitropropionic acid-induced cell death in murine corticostriatal slice cultures. Brain Res..

[9] Matthews R.T., Yang L.C., Jenkins B.G., Ferrante R.J., Rosen B.R., Kaddurah-Daouk R., Beal M.F. (1998). Neuroprotective effects of creatine and cyclocreatine in animal models of Huntington’s disease. J. Neurosci..

[10] Matthews R.T., Ferrante R.J., Klivenyi P., Yang L.C., Klein A.M., Mueller G., Kaddurah-Daouk R., Kaddurah-Daouk M.F. (1999). Creatine and cyclocreatine attenuate MPTP neurotoxicity. Exp. Neurol..

[11] Juhn M.S., Tarnopolsky M. (1998). Oral creatine supplementation and athletic performance: A critical review. Clin. J. Sport Med..

[12] Balsom P.D., Soderlund K., Sjodin B., Ekblom B. (1995). Skeletal muscle metabolism during short duration high-intensity exercise: Influence of creatine supplementation. Acta Physiol. Scand..

[13] Greenhaff P.L., Bodin K., Soderlund K., Hultman E. (1994). Effect of oral creatine supplementation on skeletal muscle phosphocreatine resynthesis. Am. J. Physiol..

[14] Gufford B.T., Sriraghavan K., Miller N.J., Miller D.W., Gu X.C., Vennerstrom J.L., Robinson D.H. (2010). Physicochemical characterization of creatine *N*-methylguanidinium salts. J. Diet. Suppl..

[15] Deldicque L., Décombaz J., Foncea H.Z., Vuichoud J., Poortmans J.R., Francaux M. (2008). Kinetics of creatine ingested as a food ingredient. Eur. J. Appl. Physiol..

[16] Dash A.K., Miller D.W., Han H.Y., Carnazzo J., Stout J.R. (2001). Evaluation of creatine transport using Caco-2 monolayers as an in vitro model for intestinal absorption. J. Pharm. Sci..

[17] Gufford B.T., Ezell E.L., Robinson D.H., Miller D.W., Miller N.J., Gu X.C., Vennerstrom J.L. (2013). pH-dependent stability of creatine ethyl ester: Relevance to oral absorption. J. Diet. Suppl..

[18] Boenzi S., Rizzo C., Ciommo V.M.D., Martinelli D., Goffredo B.M., Marca G.I., Dionisi-Vici C. (2011). Simultaneous determination of creatine and guanidinoacetate in plasma by liquid chromatography-tandem mass spectrometry (LC-MS/MS). J. Pharm. Biomed. Anal..

[19] Carling R.S., Hogg S.L., Wood T.C., Calvin J. (2008). Simultaneous determination of guanidinoacetate, creatine and creatinine in urine and plasma by un-derivatized liquid chromatography-tandem mass spectrometry. Ann. Clin. Biochem..

[20] Chanutin A. (1925). The Fate of Creatine When Administered in Man. J. Biol. Chem..

[21] Miller D.W., Augustine S., Robinson D.H., Vennerstrom J.L., Wagner J.C. (2013). Oral Bioavailability of Creatine Supplements: Insights into Mechanism and Implications for Improved Absorption. Nutrition and Enhanced Sports Performance: Muscle Biulding, Endurance, and Strength.

[22] McCall W., Persky A.M. (2007). Pharmacokinetics of creatine. Subcell. Biochem..

[23] Wyss M., Kaddurah-Daouk R. (2000). Creatine and creatinine metabolism. Physiol. Rev..

[24] Davies N.M., Takemoto J.K., Brocks D.R., Yáñez J.A. (2010). Multiple peaking phenomena in pharmacokinetic disposition. Clin. Pharmacokinet..

[25] Deminice R., Jordao A.A. (2012). Creatine supplementation reduces oxidative stress biomarkers after acute exercise in rats. Amino Acids.

[26] Harris R.C., Nevill M., Harris D.B., Fallowfield J.L., Bogdanis G.C., Wise J.A. (2002). Absorption of creatine supplied as a drink, in meat or in solid form. J. Sports Sci..

[27] Schedel J.M., Tanaka H., Kiyonaga A., Shindo M., Schutz Y. (1999). Acute creatine ingestion in human: Consequences on serum creatine and creatinine concentrations. Life Sci..

[28] Sale C., Harris R.C., Florance J., Kumps A., Sanvura R., Poortmans J.R. (2009). Urinary creatine and methylamine excretion following 4 × 5 g day^−1^ or 20 × 1 g day^−1^ of creatine monohydrate for 5 days. J. Sports Sci..

[29] Atassi N., Ratai E.M., Greenblatt D.J., Pulley D., Zhao Y.L., Bombardier J., Wallace S., Eckenrode J., Cudkowicz M., Dibernardo A. (2010). A phase I, pharmacokinetic, dosage escalation study of creatine monohydrate in subjects with amyotrophic lateral sclerosis. Amyotroph. Lateral Scler..

[30] Snow R.J., Murphy R.M. (2001). Creatine and the creatine transporter: A review. Mol. Cell. Biochem..

[31] Braissant O. (2012). Creatine and guanidinoacetate transport at blood-brain and blood-cerebrospinal fluid barriers. J. Inherit. Metab. Dis..

[32] Nash S.R., Giros B., Kingsmore S.F., Rochelle J.M., Suter S.T., Gregor P., Seldin M.F., Caron M.G. (1994). Cloning, pharmacological characterization, and genomic localization of the human creatine transporter. Recept. Channels.

[33] Sora I., Richman J., Santoro G., Wei H.B., Wang Y., Vanderah T., Horvath R., Nguyen M., Waite S., Roeske W.R. (1994). The cloning and expression of a human creatine transporter. Biochem. Biophys. Res. Commun..

[34] Salomons G.S., van Dooren S.J.M., Verhoeven N.M., Cecil K.M., Ball W.S., Degrauw T.J., Jakobs C. (2001). X-linked creatine-transporter gene (SLC6A8) defect: A new creatine-deficiency syndrome. Am. J. Hum. Genet..

[35] Tachikawa M., Tachikawa M., Fukaya M., Terasaki T., Ohtsuki S., Watanabe M. (2004). Distinct cellular expressions of creatine synthetic enzyme GAMT and creatine kinases uCK-Mi and CK-B suggest a novel neuron-glial relationship for brain energy homeostasis. Eur. J. Neurosci..

[36] Ohtsuki S., Tachikawa M., Takanaga H., Shimizu H., Watanabe M., Hosoya K., Terasaki T. (2002). The blood-brain barrier creatine transporter is a major pathway for supplying creatine to the brain. J. Cereb. Blood Flow Metab..

[37] Salomons G.S., Wyss M. (2007). Creatine and Creatine Kinase in Health and Disease: From Cell Deconstruction to System Reconstruction.

[38] Jager R., Harris R.C., Purpura M., Francaux M. (2007). Comparison of new forms of creatine in raising plasma creatine levels. J. Int. Soc. Sports Nutr..

[39] Allan G., Davis J., Dickins M., Garder I., Jenkins T., Jones H., Webster R., Westgate H. (2008). Pre-clinical pharmacokinetics of UK-453,061, a novel non-nucleoside reverse transcriptase inhibitor (NNRTI), and use of in silico physiologically based prediction tools to predict the oral pharmacokinetics of UK-453,061 in man. Xenobiotica.

[40] Bungay P.J., Tweedy S., Howe D.C., Gibson K.R., Jones H.M., Mount N.M. (2011). Preclinical and clinical pharmacokinetics of PF-02413873, a nonsteroidal progesterone receptor antagonist. Drug Metab. Dispos..

[41] De Buck S.S., Sinha V.K., Fenu L.A., Nijsen M.J., Mackie C.E., Gilissen R.A.H.J. (2007). Prediction of human pharmacokinetics using physiologically based modeling: A retrospective analysis of 26 clinically tested drugs. Drug Metab. Dispos..

[42] Jones H.M., Gardner I.B., Collard W.T., Stanley P.J., Oxley P., Hosea N.A., Plowchalk D., Gernhardt S., Lin J., Dickins M. (2011). Simulation of human intravenous and oral pharmacokinetics of 21 diverse compounds using physiologically based pharmacokinetic modelling. Clin. Pharmacokinet..

[43] Jones H.M., Dickins M., Youdim K., Gosset J.R., Attkins N., Hay T.L., Gurrell I.K., Logan Y.R., Bungay P.J., Jones B.C. (2012). Application of PBPK modelling in drug discovery and development at Pfizer. Xenobiotica.

[44] Sinha V.K., Snoeys J., Osselaer N.V., Peer A.V., Mackie C., Heald D. (2012). From preclinical to human—Prediction of oral absorption and drug-drug interaction potential using physiologically based pharmacokinetic (PBPK) modeling approach in an industrial setting: A workflow by using case example. Biopharm. Drug Dispos..

[45] Xia B., Heimbach T., He H., Lin T.H. (2012). Nilotinib preclinical pharmacokinetics and practical application toward clinical projections of oral absorption and systemic availability. Biopharm. Drug Dispos..

[46] Yamazaki S., Skaptason J., Romero D., Vekich S., Jones H.M., Tan W.W., Wilner K.D., Koudriakova T. (2011). Prediction of oral pharmacokinetics of cMet kinase inhibitors in humans: Physiologically based pharmacokinetic model versus traditional one-compartment model. Drug Metab. Dispos..

[47] Tuckfield C. (2015). First use of creatine hydrochloride in premanifest Huntington disease. Med. J. Aust..

